# Sensorless Load Torque Estimation and Passivity Based Control of Buck Converter Fed DC Motor

**DOI:** 10.1155/2015/132843

**Published:** 2015-03-29

**Authors:** S. Ganesh Kumar, S. Hosimin Thilagar

**Affiliations:** DEEE, Anna University, Chennai 600 025, India

## Abstract

Passivity based control of DC motor in sensorless configuration is proposed in this paper. Exact tracking error dynamics passive output feedback control is used for stabilizing the speed of Buck converter fed DC motor under various load torques such as constant type, fan type, propeller type, and unknown load torques. Under load conditions, sensorless online algebraic approach is proposed, and it is compared with sensorless reduced order observer approach. The former produces better response in estimating the load torque. Sensitivity analysis is also performed to select the appropriate control variables. Simulation and experimental results fully confirm the superiority of the proposed approach suggested in this paper.

## 1. Introduction

Load torque estimation of DC motor is essential in trajectory tracking control of radar, robots, motorized seat belt systems [1], and wearable exoskeletons [2]. Practically, torque is measured using either contact or noncontact type torque sensor where the latter is not economic. In [2], load torque estimation is completed in the absence of torque sensor with the knowledge of plant model inversion. Nowadays online algebraic approach [3] gains more interest in estimating the load torque of DC motor due to its fast estimation without any tuning requirement [4] as well as model inversion. For estimating the load torque, speed, armature voltage, and armature current are used as feedback variables [3, 4].

Torque can be estimated without a speed sensor and hence the number of sensors will be reduced. Therefore it is proposed to implement online algebraic load torque estimation without a speed sensor. In this scheme, speed is estimated using the feedback variables such as armature voltage and armature current through the corresponding mathematical model and hence the scheme can be termed as sensorless.

Further to regulate the speed of DC motor with and without load, passivity based control is preferred due to its robustness [3, 5–7] and stability [3, 7–9]. Due to these merits, passivity based control is used in various applications such as piezoelectric Timoshenko beam [10], bilateral teleoperation [11], and flight control design [12].

In addition to the above mentioned merits, passivity based control law uses most sensitive variable [13] which makes the controller more effective in comparison with other controllers like proportional-integral controller [8, 9].

In passivity based control, exact tracking error dynamics passive output feedback method is preferred in comparison with energy shaping and damping injection method due to the absence of controller states computation [5]. This motivates the authors to implement exact tracking error dynamics passive output feedback control for DC motor in sensorless mode. Previously, boost converter [3, 13] and boost rectifier [4] are used as converter for DC motor. In continuation of this, a simple differentially flat buck converter is selected in this present work.

This paper is organised as follows. Modeling and control of buck converter fed DC motor is presented in Section 2. Sensitivity analysis for the selection of control variables is discussed in Section 3. Sensorless load torque estimation is dealt with in Section 4. Simulation and hardware results are explained in Section 5. Conclusions and the future scope for the work are discussed in Section 6.

## 2. Modelling and Control of Buck Converter Fed DC Motor

**Figure 1:**
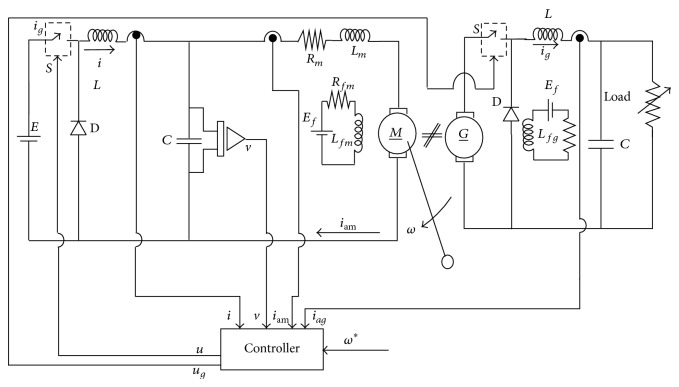
Sensorless load torque estimation of buck converter fed DC motor.

Implementation of sensorless load torque estimation for buck converter fed DC motor is shown in Figure 1 in which *ω* ^∗^ is considered as desired speed. Feedback signals *v*,  *i*_am_ are used for load torque estimation in sensorless mode configuration and “*i*” is used for exact tracking error dynamics passive output feedback control implementation. For the implementation of speed regulation using exact tracking error dynamics passive output feedback control, resistive load [3, 4] is not added with DC motor load so that power loss can be avoided. Earlier, resistive load was included to satisfy dissipation matching condition [14], and in the present case it is assessed using LaSalle's invariance principle [15].

Buck converter and DC generator coupled with DC motor (Figure 1) are used for realizing load torque such as constant, fan type, propeller type, and unknown load torque. Control input “*u*” is responsible for speed control and *u*_*g*_ is used for realizing the abovesaid load torques. In order to implement exact tracking error dynamics passive output feedback control, model for the buck converter fed DC motor is modified into energy management structure, and it is presented in the following:

(1) $$\begin{array}{c} \dot{x}\left(t\right)=\left[J-R\right]{\left(\frac{\partial H\left(x\right)}{\partial x}\right)}^{T}+bu+\epsilon , \end{array}$$

(2) $$\begin{array}{c} {\left(\frac{\partial H\left(x\right)}{\partial x}\right)}^{T}=Mx. \end{array}$$

Matrices *M*, *b*, *ϵ*, *x*(*t*), *J*, and *R* are given by

(3) $$\begin{array}{c} M=\left[\begin{array}{cccc} L & 0 & 0 & 0\\ 0 & C & 0 & 0\\ 0 & 0 & L_{m} & 0\\ 0 & 0 & 0 & J \end{array}\right],\;\;b^{T}=\left[\begin{array}{cccc} \frac{E}{L} & 0 & 0 & 0 \end{array}\right];\\ \epsilon^{T}=\left[\begin{array}{cccc} 0 & 0 & 0 & \frac{{-T}_{L}}{J} \end{array}\right];\;\;x{\left(t\right)}^{T}=\left(i,v,i_{\text{am}},\omega \right),\\ J=\left[\begin{array}{cccc} 0 & \frac{-1}{LC} & 0 & 0\\ \frac{1}{LC} & 0 & \frac{-1}{L_{m}C} & 0\\ 0 & \frac{1}{L_{m}C} & 0 & \frac{-k}{JL_{m}}\\ 0 & 0 & \frac{k}{JL_{m}} & 0 \end{array}\right];\;\;R=\left[\begin{array}{cccc} 0 & 0 & 0 & 0\\ 0 & 0 & 0 & 0\\ 0 & 0 & \frac{R_{m}}{L_{m}^{2}} & 0\\ 0 & 0 & 0 & \frac{B}{J^{2}} \end{array}\right], \end{array}$$

where *k*: EMF constant, *L*: buck converter inductance, *C*: buck converter capacitance, *R*_*m*_: motor armature resistance, *L*_*m*_: motor armature inductance, *u*: average control input, *i*: input current, *v*: armature voltage or converter output voltage, *ω*: angular velocity of the motor shaft (2*πN*/60), *T*_*L*_: load torque, *i*_am_: motor armature current, *N*: speed of the motor shaft, *J*: motor inertia, *B*: frictional coefficient, and *E*: Input voltage.

The matrix “*J*” is independent of “*u*” and it is of skew-symmetry in nature. Matrix *R* is symmetric and positive semidefinite; that is, *R* ^*T*^ = *R* ≥ 0. To regulate the speed of DC motor, exact tracking error dynamics passive output feedback control is essential and it is derived based on the error stabilisation dynamics [15] which is presented in the following section.

### 2.1. Exact Tracking Error Dynamics Passive Output Feedback Control Design

The main objective of the present work is to regulate the speed of a DC motor under no-load and load conditions for a given or desired speed profile (*ω* ^∗^). For this speed profile, it is assumed that a state reference trajectory satisfies the desired open loop dynamics and it is given by

(4) $$\begin{array}{c} {\dot{x}}^{*}\left(t\right)=\left(J-R\right){\left(\frac{\partial H\left(x^{*}\right)}{\partial x^{*}}\right)}^{T}+bu^{*}+\epsilon . \end{array}$$

In order to satisfy these dynamics, a linear time-varying average incremental passive output feedback controller is simply derived from [14] and it is given as follows:

(5) $$\begin{array}{c} u-u^{*}=-\gamma {\overset{ˇ}{b}}^{T}{\left(\frac{\partial H\left(e\right)}{\partial e}\right)}^{T}=-\gamma {\overset{ˇ}{b}}^{T}Me=-\gamma \frac{E}{L}\left(i-i^{*}\right), \end{array}$$

where *u* ^∗^: desired control input, *i* ^∗^: desired inductor current, and *γ*: damping injection coefficient >0:

(6) $$\begin{array}{c} \overset{ˇ}{b}=\left[b+\left(\frac{\partial J\left(u\right)}{\partial u}\right)\left[{\left(\frac{\partial H\left(x^{*}\right)}{\partial x^{*}}\right)}^{T}\right]\right]. \end{array}$$

Now, error dynamics of system [14] is given by

(7) $$\begin{array}{c} \dot{e}=J{\left(\frac{\partial H\left(e\right)}{\partial e}\right)}^{T}-\tilde{R}{\left(\frac{\partial H\left(e\right)}{\partial e}\right)}^{T}. \end{array}$$

With the skew symmetry nature of *J*, $\dot{H}\left(e\right)$ is given by

(8) $$\begin{array}{c} \dot{H}\left(e\right)=-\left(\frac{\partial H\left(e\right)}{\partial e}\right)\tilde{R}{\left(\frac{\partial H\left(e\right)}{\partial e}\right)}^{T}\leq 0. \end{array}$$

Equation (8) is negative semidefinite, as dissipation matching condition ($\tilde{R}$) is not satisfied which is given as follows:

(9) R~=R+b+∂J∂u∂Hx∗∂x∗T ·γb+∂J∂u∂Hx∗∂x∗TT,R~=γE2L2000000000RmLm20000BJ2≥0.

As $\tilde{R}\geq 0$, LaSalle's theorem [15] is used to establish global asymptotic stability of the origin of the tracking error space. In order to verify LaSalle's theorem, substituting the necessary expressions in (8), (10) is obtained:

(10) $$\begin{array}{c} \dot{H}\left(e\right)=-\left(\gamma e_{1}^{2}E^{2}+e_{3}^{2}R_{m}+e_{4}^{2}B\right). \end{array}$$

When $\dot{H}\left(e\right)=0$, if errors *e*_1_, *e*_3_, and *e*_4_ become zero, then ${\dot{e}}_{1}={\dot{e}}_{3}={\dot{e}}_{4}=0$. On substituting these values in (10), then *e*_2_ = 0. Hence, the error dynamics of the system is globally asymptotically stable and it satisfies LaSalle's invariance principle. Due to the bounded nature of control input, system stability is not a global one.

Following are the steps involved in implementing the speed regulation of DC motor using exact tracking error dynamics passive output feedback control.

When the speed is to be increased from one value to another, it requires change in energy. Based on this, reference profiles of control input, voltage, and inductor current under no-load conditions and load conditions are modified. Under load conditions, SAA and SROO methods are used for load torque estimation. Based on the modification in the reference profiles, error in inductor current and control input is calculated. With this error, new control input is obtained which modifies the energy in the system so that the speed is increased. When the speed increases, error will become zero, and thus the system becomes stable. Convergence rate of error can be modified by introducing damping injection coefficient.

Control function (*u*) in (5) clearly indicates the absence of derivative term which makes the controller simpler. This confirms the selection of exact tracking error dynamics passive output feedback control in the present work. When “*u*” semiglobally stabilizes to *u* ^∗^ output voltage “*v*,” current of the buck converter “*i*” and speed “*ω*” of the motor will reach the corresponding steady state values. These steady state values are nothing but the reference values to achieve speed regulation without or with load conditions. These references are derived by differential parameterisations of (1), and the derived expressions in terms of desired speed *ω* ^∗^ and load torque (${\hat{T}}_{L}$) are given in (11)–(14). As the buck converter fed DC motor is differentially flat [14], reference profiles are obtained easily:

(11) $$\begin{array}{c} v^{*}\left(t\right)=a_{3}\ddot{\omega^{*}}+a_{4}\dot{\omega^{*}}+a_{5}\omega^{*}+\frac{{\hat{T}}_{L}R_{m}}{k}, \end{array}$$

(12) $$\begin{array}{c} i_{\text{am}}^{*}\left(t\right)=a_{1}\dot{\omega^{*}}+a_{2}\omega^{*}+\frac{\hat{T_{L}}}{k}, \end{array}$$

(13) $$\begin{array}{c} i^{*}\left(t\right)=a_{6}\dddot{\omega^{*}}+a_{7}\ddot{\omega^{*}}+a_{8}\dot{\omega^{*}}+a_{2}\omega^{*}+\frac{\hat{T_{L}}}{k}, \end{array}$$

(14) $$\begin{array}{c} u^{*}=\frac{v^{*}}{E}, \end{array}$$

where

(15) $$\begin{array}{c} a_{1}=\frac{J}{k};\;a_{2}=\frac{B}{k};\;a_{3}=\frac{JL_{m}}{k};\;a_{4}=\frac{BL_{m}+JR_{m}}{k};\\ a_{5}=\left(\frac{BR_{m}}{k}+k\right);\;a_{6}=\frac{CJL_{m}}{k};\;a_{7}=C\left(\frac{BL_{m}+JR_{m}}{k}\right);\\ a_{8}=\left(\frac{CBR_{m}}{k}+Ck+\frac{J}{k}\right). \end{array}$$

Desired armature current and armature voltage expressions are used for identifying sensitive variable and it is presented in Section 3.

In order to define the speed trajectory, Bezier polynomial [14] of tenth order is used. Previously, Bezier polynomials are used in the design of smooth surfaces in automobile parts as well as in graphic designing. The desired Bezier polynomial for speed is given by

(16) ω∗t=ωini for  t<tini;=ωfin for  t>tfin;=ωini+∅ωfin−ωini for  other  values  of  “t”,

where the expression for *∅* is given below:

(17) $$\begin{array}{c} \emptyset =252g^{5}-1050g^{6}+1800g^{7}-1575g^{8}+700g^{9}-126g^{10}, \end{array}$$

where

(18) $$\begin{array}{c} g=\left(\frac{t-t_{\text{ini}}}{t_{\text{fin}}-t_{\text{ini}}}\right). \end{array}$$

Thus the desired references for inductor current, armature current, and armature voltage are obtained and based on the inductor current reference profile; exact tracking error dynamics passive output feedback control can be implemented under no-load and load conditions. Under load conditions, load torque estimation is required for the modification of inductor current reference.

## 3. Sensitivity Analysis of Exact Tracking Error Dynamics Passive Output Feedback Control

Sensitivity analysis is used in power systems and power electronics [16, 17] for various aspects such as to investigate the effect of parameter variation in the system or for formulating the steady state of a system. Untill date sensitivity theory is successfully implemented for finding the system stability [18] optimal design [19]. Hence it is proposed to investigate the sensitivity analysis of speed of the DC motor to the state variables of buck converter fed DC motor through frequency response analysis. Further sensitivity analysis is used to identify the appropriate control variable which is inherently used in exact tracking error dynamics passive output feedback control of converter fed DC motor.

Bode plots are obtained between the variables such as speed and armature current, armature voltage, and inductor current, respectively, for the load, varying from zero to 100% of the rated value. Frequency domain expressions for speed in terms of armature current, armature voltage, inductor current, and load torque are obtained through differential parameterisations, and the derived expressions are presented in (19). Specifications mentioned in Table 1 are used for performing sensitivity analysis:

(19) ωs=iasa1s+a2−TLka1s2+a2s,ωs=vsa3s2+a4s+a5−TLRm/ka3s3+a4s2+a5s,ωs=isa6s3+a7s2+a8s+a2 −TL1/ka6s4+a7s3+a8s2+a2s.

**Figure 2:**
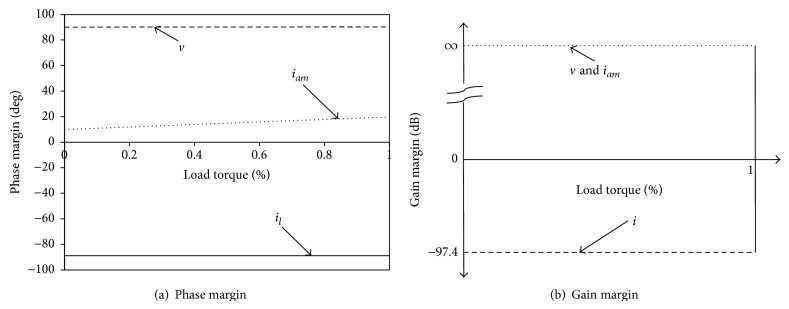
Sensitivity analysis of passivity based control.

Figure 2 indicates the gain margin and phase margin characteristics of buck converter fed DC motor output variable speed to state variables such as armature current, armature voltage, and inductor current. Figure 2 reveals that gain margin values remain constant for various values of load torque. Gain and phase margin for inductor current is negative which makes inductor current more sensitive and it is inherently used in passivity based control law (5).

From the above discussion, it is concluded that due to the selection of sensitive variable in passivity based control law, its dynamic response is better than proportional integral control [7, 8]. Thus sensitivity property is verified for the state variables inductor current, armature voltage, and armature current.

## 4. Sensorless Load Torque Estimation

**Figure 3:**
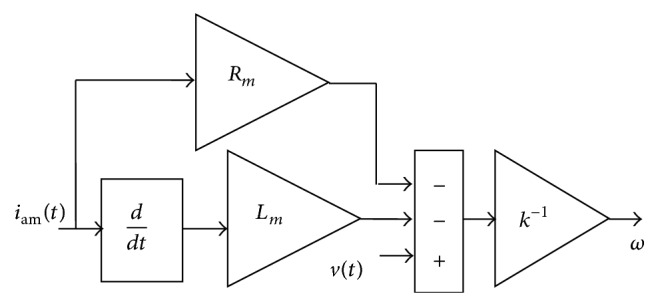
Sensorless scheme.

This section deals with the online estimation of load torque using algebraic estimation and reduced order observer techniques. Due to the advantages mentioned in [3, 4], algebraic estimation technique is used for load torque estimation and, in continuation of that, sensorless approach is proposed for algebraic estimation of load torque which can be derived from the dynamic model of DC motor [4] instead of from energy principles [3]. The basic assumption is that the uncertain load torque is piecewise constant. In order to implement sensorless scheme, estimation is done from armature voltage and armature current and it is shown in Figure 3. Sensorless online algebraic approach (SAA) and sensorless reduced order observer (SROO) approach are completed [13] and estimated torques using these approaches are given in (20) and (21):

(20) τL=2t−ti2 ·J∫tit1kvt−Rmiam(t)−Lmdiam(t)dtdτkk −Jt−ti1kvt−Rmiamt−Lmdiamtdtkk +k∫titt−tiiamτdτkk −B∫titt−tikkk   ·1kvt−Rmiamt−Lmdiamtdtdτ.

Estimation of load torque using SAA needs updating of load torque for every 0.003 seconds. Past torque values are neglected and the response time for estimating the torque becomes faster:

(21) dξdt=−λξ+λkiamt+λλJ−B ·1kvt−Rmiamt−Lmdiamtdt,

where *λ* > 0 is tuning gain.

From (20) and (21), it can be concluded that speed sensor is not required for load torque estimation using SAA and SROO. Thus SAA and SROO are discussed in detailed manner and it is observed that, in order to compare SAA and SROO, simulation study and real-time implementation was completed and it is presented in the next section.

## 5. Results

**Figure 4:**
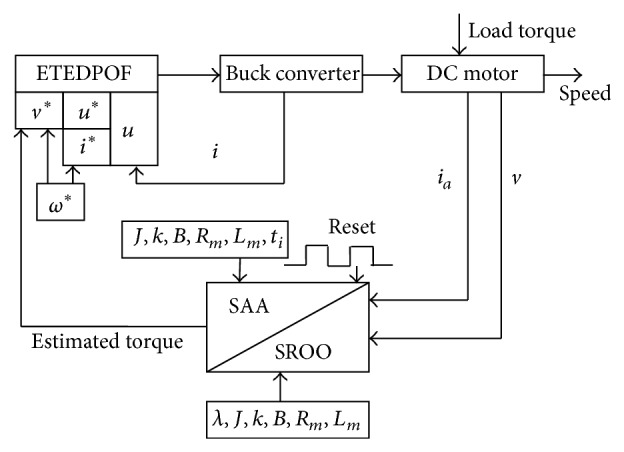
Flow diagram for ETEDPOF implementation.

Both SROO and SAA are implemented for buck converter fed DC motor in simulation and real-time for different load conditions with different speed profiles. MATLAB SIMULINK is used for simulation and the results are compared with those of hardware implementation. State constructors are used for simulation. Exact tracking error dynamics passive output feedback (ETEDPOF) control is implemented by following the flow diagram shown in Figure 4 and it shows that feedback signals such as inductor current (*i*), armature current (*i*_am_), and armature voltage (*v*) are required for feedback control under loading conditions.

From the desired speed *ω* ^∗^ and the estimated torque ${\hat{\tau }}_{L}$, desired voltage *v* ^∗^ and inductor current *i* ^∗^ values are obtained using (11) and (13). From the values of *v* ^∗^,  *u* ^∗^ is derived using (14). *u* ^∗^ and *i* ^∗^ are used along with the feedback signal “*i*” for obtaining the value of control input “*u*” mentioned in (5). This control input is applied to the switch of buck converter so that speed is regulated. The value of damping injection (*γ*) is taken as 0.125 so that noise amplification is minimal [3].

Under no-load and constant load torque conditions, buck converter inductor current alone is required for speed regulation. When load is applied on the motor side, the updated torque value is used for changing the reference values of armature current, inductor current, and armature voltage. The specifications for the system of interest are given in Table 1. For convenience, per unit conversion is adopted in this work.

**Figure 5:**
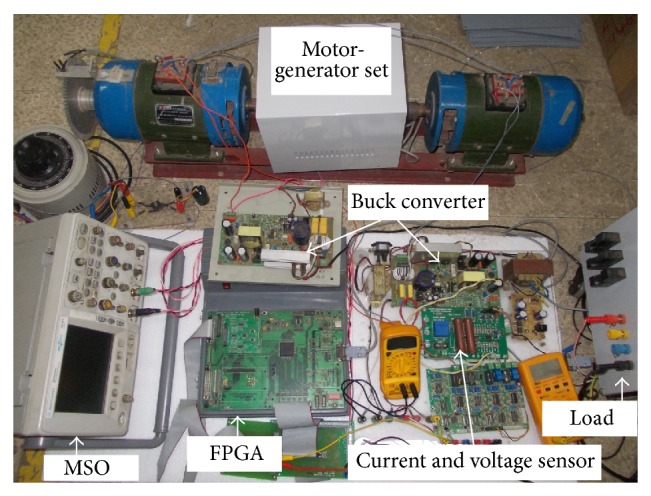
Experimental setup.

The experimental setup shown in Figure 5 is used for the implementation of SAA and SROO. This setup includes buck converter, DC motor, DC generator, controller, and the necessary sensors with signal conditioning circuits. The speed output is measured using phototransistor (MOC 7811) and encoder disc.

The advantage of coupling a DC generator is that it can be used for realizing any type of load torque which is achieved by adjusting generator current in proportion to the torque-speed profile. Constant torque in the generator side is obtained in such a way that torque never exceeds rated value. No-load condition is realized when the generator is not connected with load.

LTS 25-NP is used as current sensor. The machine parameters are obtained by following standard test procedures. Buck converter and controller are used for controlling the speed of DC motor under armature control method. Desired PWM pulses for the closed loop operation are obtained using system generator, Spartan-3A DSP Trainer Kit, which comprises Xilinx XC3SD1800A–FG676-4 Spartan 3A DSP FPGA and allied accessories. The clock frequency is 20 MHz and the sampling frequency of ADC is 2 MHz. For estimating the load torque using SAA, period of rest and reset time are taken as 0.003 seconds and 0.03 seconds, respectively. In order to obtain speed regulation of DC motor using FPGA, all the parameters should be properly quantized. The feedback of inductor current, armature current, and armature voltage are fed to the FPGA ADC channels through the necessary signal conditioning circuits. The analog outputs such as load torque, armature current, inductor current, speed, and armature voltage are taken from DAC channels of FPGA kit.

### 5.1. Discussions

Simulation and hardware implementation for buck converter fed DC motor is completed for 12 seconds. Based on the free acceleration, change of speed reference, and load torque conditions, total operation is divided into two broad categories: servo and regulatory control mode. In servo control mode, speed control of buck converter fed DC motor is done for free acceleration characteristics and at different constant load torques. In regulatory control mode, speed control is achieved up to the rated speed along with two load torque estimation schemes, namely, SAA, SROO, and both are compared. In SROO, based on the value of tuning constant, it is classified into SROO1 and SROO2 for which *λ* value is equal to 5 and 10, respectively. Servo and regulatory control modes are explained below.

**Figure 6:**
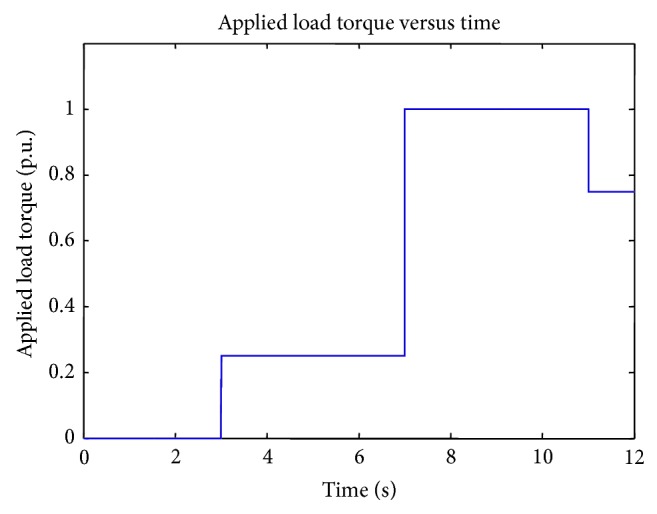
Applied load torque.

**Figure 7:**
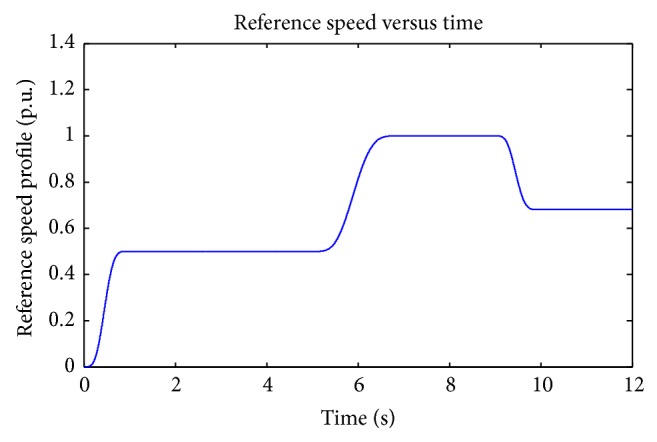
Speed reference.

**Figure 8:**
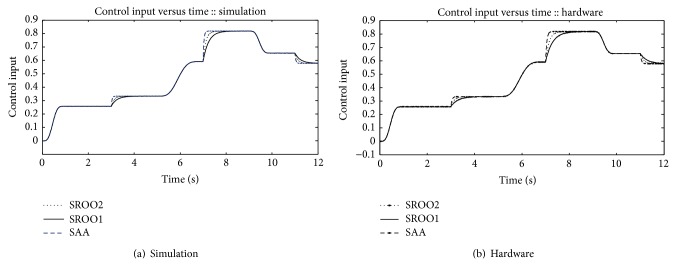
Control input.

**Figure 9:**
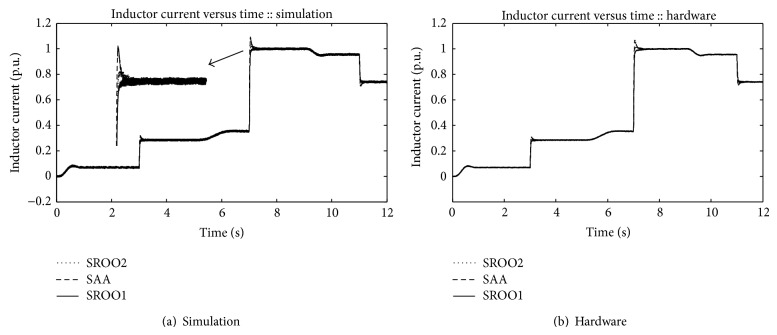
Inductor current.

**Figure 10:**
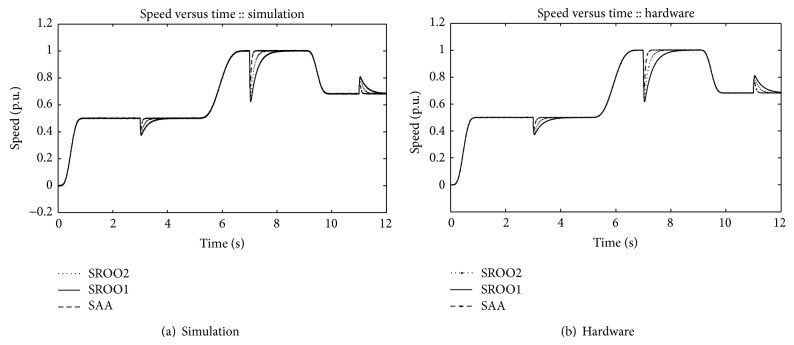
Speed.

**Figure 11:**
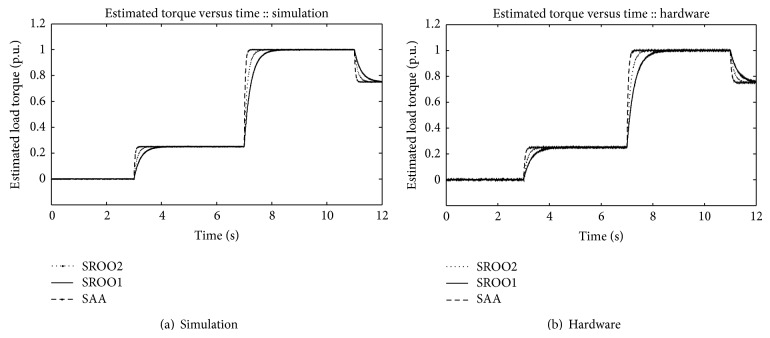
Estimated torque.

**Figure 12:**
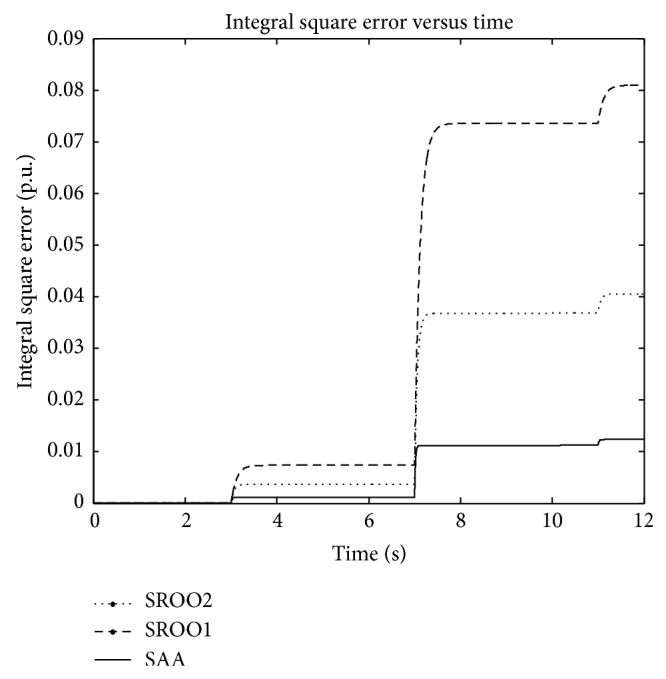
Integral square error.

**Figure 13:**
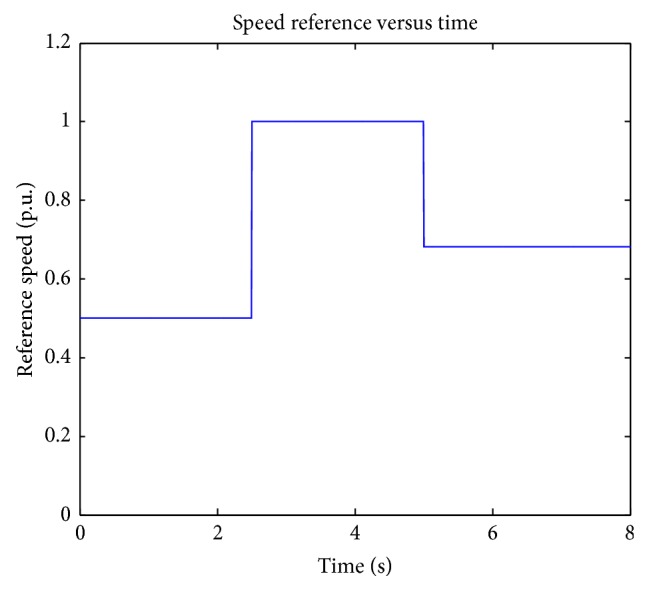
Speed reference: *T*_*L*_ *αω*.

**Figure 14:**
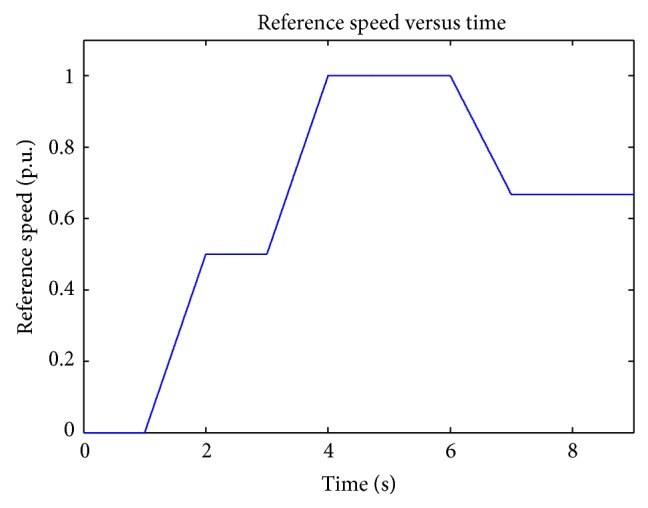
Speed reference: *T*_*L*_ *αω* ^2^.

**Figure 15:**
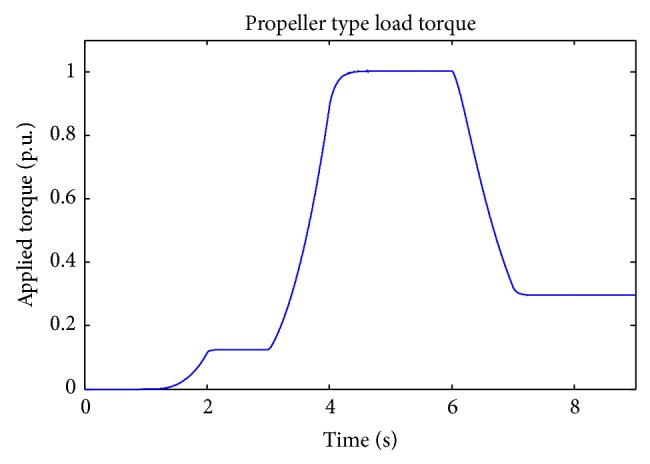
*T*_*L*_ *αω* ^3^.

**Figure 16:**
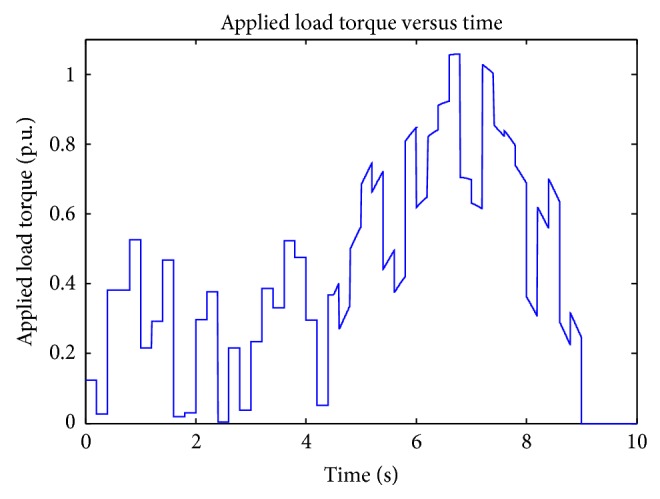
Unknown type load torque.

**Figure 17:**
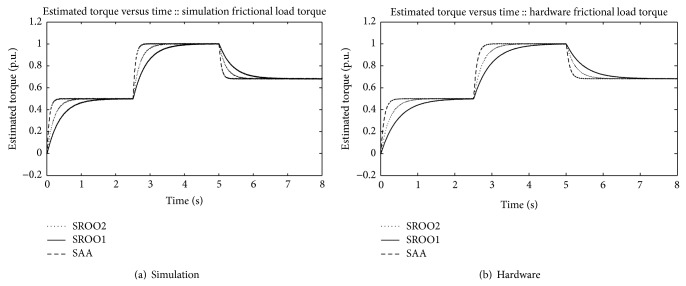
Estimated torque.

**Figure 18:**
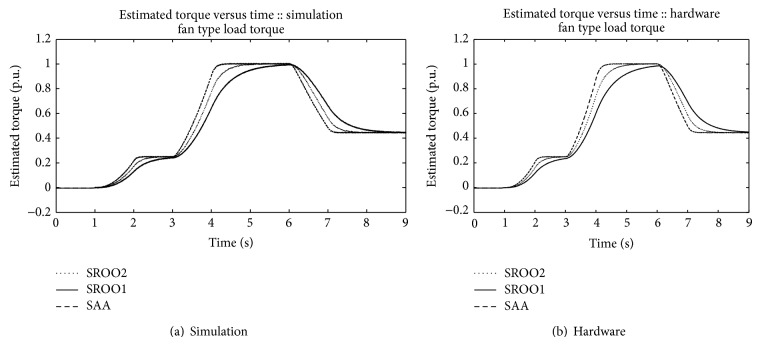
Estimated torque: *T*_*L*_ *αω* ^2^.

**Figure 19:**
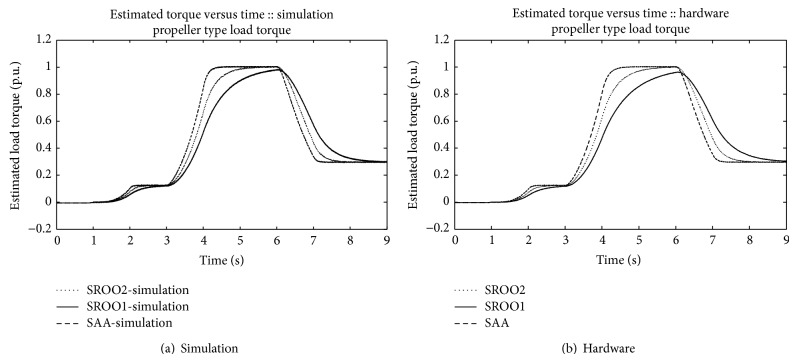
Estimated torque: *T*_*L*_ *αω* ^3^.

**Figure 20:**
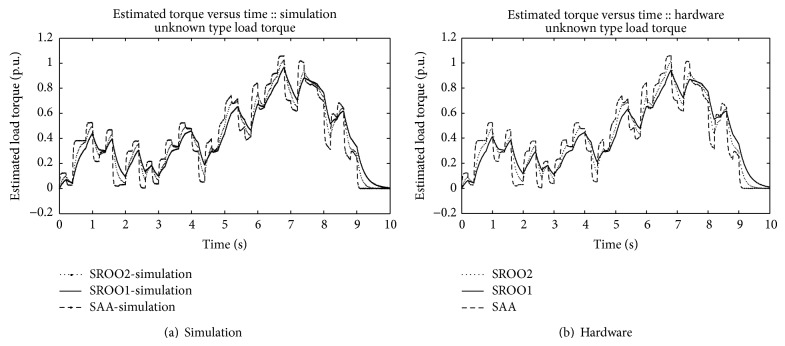
Estimated torque.

**Figure 21:**
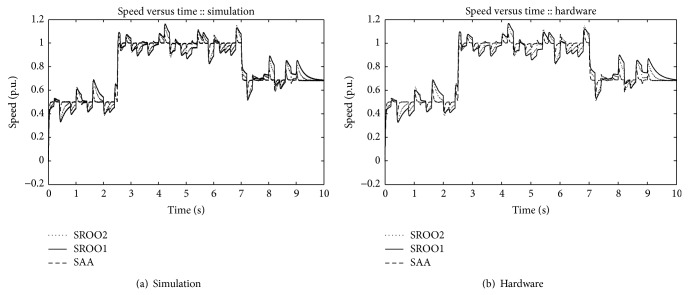
Speed-unknown load torque.

Hardware and simulation results are shown from Figures 6 to 21. Simulation and hardware results are obtained separately and data files from simulation and hardware are exported to MATLAB for plotting. Responses for SAA and SROO approaches are shown separately. From the plots, it is observed that under servo control, control input variation is similar for SAA and SROO in both hardware and simulation. In hardware implementation, ripple content is more in comparison with simulation study due to switching of the devices. In simulation, ideal components are used. Hence, simulation performance is slightly better than hardware implementation.

Features of exact tracking error dynamics passive output feedback control, SAA, and SROO methods are analysed through the results obtained and they are presented below.

Figure 6 shows the load torque applied in the separately excited DC motor. Rated load torque of the DC motor is 4.75 Nm. Applied load torque values are 0.25 p. u., 1.00 p. u., and 0.75 p. u. at 3rd, 7th, and 11th seconds.

In order to obtain the speed regulation of DC motor, reference profile for speed is essential and it is shown in Figure 7. These speed profiles are used in radar tracking and in robotics. Smooth variation in speed reference is achieved using Bezier polynomial of tenth order shown in (16).

Exact tracking error dynamics passive output feedback control for buck converter fed DC motor is obtained using (5). Based on the reference profiles for inductor current, control input, and inductor current feedback, speed regulation is achieved using the modified control input shown in Figure 8. Under load conditions, SAA dominates SROO in modifying the control input which is clearly found in Figure 8. As armature voltage varies linearly with control input, control input alone is presented here.

Inductor current variation for the exact tracking error dynamics passive output feedback control is shown in Figure 9 and under servo control operation (between 0 and 1 second, 5 and 6 seconds, and 9 and 10 seconds) exhibits no overshoot beyond rated current which confirms the implementation of soft starting. Under regulatory control, overshoot/undershoot values in SAA are higher than SROO which is due to the sudden change in speed of DC motor and it can be seen in Figure 9 (zoomed view). As inductor current variation is similar to armature current variation, inductor current response alone is presented.

Speed variation under servo and regulatory control operations is shown in Figure 10. Under servo control, speed is controlled using exact tracking error dynamics passive output feedback technique. Under regulatory control operation, speed is regulated using exact tracking error dynamics passive output feedback control with SAA and SROO. Both regulatory and servo control operations are explained below.

At 3rd second, 25% of load torque is applied in the DC motor. Desired speed is 0.50 p. u. during this interval. Here SAA produces an undershoot of 0.095 p. u. and 0.09 p. u. in simulation and hardware, respectively. On the other hand, SROO1 and SROO2 produce an undershoot of 0.12 p. u. and 0.118 p. u. and 0.128 p. u. and 0.124 p. u. in simulation and hardware, respectively. Settling times for SAA, SROO2, and SROO1 in simulation study are 0.20, 0.50, and 0.90 seconds, respectively. In hardware implementation, settling times are 0.25, 0.60, and 1.00 seconds, respectively.

Figure 11 shows the estimated torque using SAA and SROO methods; both methods are tested for the step change in load torque values of 0.25 p. u., 1.00 p. u., and 0.75 p. u. From Figure 11, the following points are inferred.

At 3rd second, load torque of value 0.25 p. u. is applied. SAA estimates the load torque in 0.20 and 0.30 seconds in simulation and hardware implementation. SROO2 takes 0.70 and 0.60 and 0.80 and 0.70 seconds, respectively, whereas SROO1 takes 1.20 and 1.40 seconds in simulation and hardware implementation under both loading conditions.

Due to the fast estimation of load torque using SAA, control input, armature voltage, and armature currents are changing at a faster rate in comparison with SROO with lesser integral square error (Figure 12). Thus, the superiority of SAA is verified with constant load torque.

In continuation of this, SAA is tested against *T*_*L*_ *αω* (frictional), *T*_*L*_ *αω* ^2^ (fan type), *T*_*L*_ *αω* ^3^ (propeller type), and unknown load torque by using the setup shown in Figure 5. An example for unknown load torque is that control of motor boat under turbulence condition. Results are obtained satisfactorily (Figures 13, 14, 15, 16, 17, 18, 19, 20, and 21).

Comparison between SAA and SROO is shown in Table 2. From the results, and Table 2, it is concluded that exact tracking error dynamics passive output feedback control and SAA perform better than exact tracking error dynamics passive output feedback control and SROO for all types of load torque conditions.

## 6. Conclusion

Using the exact tracking error dynamics passive output feedback control law soft start, servo and regulatory control operations of DC motor drive have been carried out. Speed tracking profile is stabilised for no-load and load conditions in the differentially flat system. As the derived controller makes the energy of the error dynamics negative semidefinite, LaSalle's theorem is established for assessing the stability of buck converter fed DC motor. Due to the flatness behaviour of this system, reference trajectories are easily obtained.

Two load torque estimation methods, SAA and SROO, were investigated through simulation and experiment for various load torques. From the results, it is observed that SAA performs better than SROO. This is due to the following facts:

- Initial condition value of load torque can be set to any arbitrary value by selecting the reset time. At each reset time past calculated torque values are omitted and the present values are updated.
- SAA does not require tuning. This SAA can be used without any change for any drive system.

Hence, it can be confirmed that SAA is capable of estimating any type of load torque in a fourth order differentially flat system, buck converter fed DC motor. This approach can be extended to other converters too.

## Figures and Tables

**Table 1:** Specifications for buck converter fed DC motor.

S. number	Buck converter		DC motor armature side	
	Symbol	Value	Symbol	Value
1	*L*	2.769 mH	*P*_*o*_	1 HP
2	Rated current	6 A	Supply voltage	180 Volt (base value)
3	*C*	440.1 *μ*F	*I*_am_	5.1 A (base value)
4	Switching frequency	32 KHz	*N*	1500 RPM (base value)
5	DC supply voltage	220 V	*L*_*m*_	111.6 mH
**DC motorfield side**			*R*_*m*_	6.1 Ω
6	*R*_*f*_	696.1 Ω	*L*_af_	3.44 H
7	*L*_*f*_	25.023 H	*J*	3.4*e* − 3 kg∗m^2^
8	*E*_*f*_	180 V	*B*	2.7*e* − 3 Nm/rad
9			Torque	4.75 Nm (base value)

**(a):** 

S. number	Time (seconds)	*ω* ^∗^ (p. u.)	*T*_*L*_ (p. u.)	Speed settling time (seconds)		
				SROO1	SROO2	SAA
	(a) Constant load torque					
1	3	0.500	0.25	0.90	0.50	0.20
2	7	1.000	1.00	1.20	0.60	0.20
3	11	0.675	0.75	1.10	0.50	0.20
				**Load torque estimation time (seconds)**		
4	3	0.500	0.25	1.00	0.50	0.20
5	7	1.000	1.00	1.20	0.70	0.20
6	11	0.675	0.75	1.20	0.60	0.20

	(b) Frictional load torque					
				**Speed settling time (seconds)**		
7	0.0–2.5	0.500	0.50	1.4	0.78	0.38
8	2.5–5.0	1.000	1.00	1.4	0.78	0.38
9	5.0–8.0	0.682	0.682	1.4	0.78	0.38
				**Load torque estimation time (seconds)**		
10	0.0–2.5	0.500	0.50	1.4	0.7	0.01
11	2.5–5.0	1.000	1.00	1.4	0.7	0.01
12	5.0–8.0	0.682	0.682	1.4	0.7	0.01

	(c) Fan type load torque					
				**Speed settling time (seconds)**		
13	1.0–2.0	Linear variation	Squared of speed	0.8	0.4	0.20
14	3.0–6.0			1.5	0.8	0.25
15	6.0–9.0			1.2	0.4	0.20
				**Load torque estimation time (seconds)**		
16	1.0–2.0	Linear variation	Squared of speed	1.0	0.2	0.01
17	3.0–6.0			2.0	0.4	0.01
18	6.0–9.0			2.0	0.4	0.01

	(d) Propeller type load torque					
				**Speed settling time (seconds)**		
19	1.0–3.0	Linear variation	Cube of speed	0.7	0.325	0.15
20	3.0–6.0			1.8	1.000	0.30
21	6.0–9.0			1.5	0.600	0.25
				**Load torque estimation time (seconds)**		
22	1.0–3.0	Linear variation	Cube of speed	1.0	0.5	0.2
23	3.0–6.0			2.2	1.5	0.5
24	6.0–9.0			2.0	1.0	0.5

**(b):** 

S. number	Time (seconds)	*ω* ^∗^ (p. u.)	*T*_*L*_ (p. u.)	Speed settling time (seconds)		
				SROO1	SROO2	SAA
	Constant load torque					
1	3	0.500	0.25	1.00	0.60	0.25
2	7	1.000	1.00	1.40	0.80	0.30
3	11	0.675	0.75	1.20	0.60	0.30
				**Load torque estimation time (seconds)**		
4	3	0.500	0.25	1.20	0.60	0.30
5	7	1.000	1.00	1.40	0.80	0.30
6	11	0.675	0.75	1.40	0.70	0.30

	Frictional load torque					
				**Speed settling time (seconds)**		
7	0.0–2.5	0.500	0.50	1.6	0.85	0.44
8	2.5–5.0	1.000	1.00	1.6	0.85	0.44
9	5.0–8.0	0.682	0.682	1.6	0.85	0.44
				**Load torque estimation time (seconds)**		
10	0.0–2.5	0.500	0.50	1.5	0.8	0.02
11	2.5–5.0	1.000	1.00	1.5	0.8	0.02
12	5.0–8.0	0.682	0.682	1.5	0.8	0.02

	Fan type load torque					
				**Speed settling time (seconds)**		
13	1.0–2.0	Linear variation	Squared of speed	1.0	0.6	0.24
14	3.0–6.0			2.0	1.0	0.40
15	6.0–9.0			1.4	0.6	0.24
				**Load torque estimation time (seconds)**		
16	1.0–2.0	Linear variation	Squared of speed	1.1	0.3	0.02
17	3.0–6.0			2.2	0.6	0.02
18	6.0–9.0			2.2	0.6	0.02

	Propeller type load torque					
				**Speed settling time (seconds)**		
19	1.0–3.0	Linear variation	Cube of speed	0.8	0.375	0.20
20	3.0–6.0			2.0	1.250	0.40
21	6.0–9.0			1.8	0.700	0.3
				**Load torque estimation time (seconds)**		
22	1.0–3.0	Linear variation	Cube of speed	1.2	0.6	0.3
23	3.0–6.0			2.4	1.6	0.6
24	6.0–9.0			2.2	1.2	0.6
